# Sex-differences in reasons for non-participation at recruitment: Geelong Osteoporosis Study

**DOI:** 10.1186/1756-0500-6-104

**Published:** 2013-03-18

**Authors:** Shikha Markanday, Sharon L Brennan, Haslinda Gould, Julie A Pasco

**Affiliations:** 1 Department of Medicine, Barwon Health, Geelong, VIC, 3220, Australia; 2 Epidemiology Unit for Musculoskeletal and Metabolic Disorders (Epi-UMMD), School of Medicine, Deakin University, PO Box 281, Geelong, VIC, 3220, Australia; 3 Department of Medicine, NorthWest Academic Centre, The University of Melbourne, Sunshine Hospital, 176 Furlong Road, St Albans, VIC, 3021, Australia; 4 Australian Institute for Musculo-Skeletal Science, NorthWest Academic Centre, The University of Melbourne, St Albans, VIC, 3021, Australia

**Keywords:** Non-participation, Recruitment, Sex-differences, Population-based, Cohort study, Epidemiology

## Abstract

**Background:**

Understanding reasons for non-participation in health studies can help guide recruitment strategies and inform researchers about potential sources of bias in their study sample. Whilst there is a paucity of literature regarding this issue, it remains highly plausible that men and women may have varied reasons for declining an invitation to participate in research. We aimed to investigate sex-differences in the reasons for non-participation at baseline of the Geelong Osteoporosis Study (GOS).

**Methods:**

The GOS, a prospective cohort study, randomly recruited men and women aged 20 years and over from a region in south-eastern Australia using Commonwealth electoral rolls (2001–06 and 1993–97, respectively). Reasons for non-participation (n=1,200) were documented during the two recruitment periods. We used the Pearson’s chi squared test to explore differences in the reasons for non-participation between men and women.

**Results:**

Non-participation in the male cohort was greater than in the female cohort (32.9% vs. 22.9%; p<0.001). Overall, there were sex-differences in the reasons provided for non-participation (p<0.001); apparent differences related to time constraints (men 26.3% vs. women 10.4%), frailty/inability to cope with or understand the study (men 18.7% vs. women 30.6%), and reluctance over medical testing (men 1.1% vs women 9.9%). No sex-differences were observed for non-participation related to personal reason/disinterest, and language- or travel-related reasons.

**Conclusions:**

Improving participation rates in epidemiological studies may require different recruitment strategies for men and women in order to address sex-specific concerns about participating in research.

## Background

Non-participation at recruitment of epidemiological studies has important implications. It has the potential to introduce selection bias: systematic groups of people may be more inclined to decline participation than others in a way which may be correlated with the variables under investigation [1]. While selection bias due to non-participation may have only a minor impact on the validity of findings from the longitudinal phase of cohort studies, there is potential for greater impact on interpretation of baseline data analysed cross-sectionally. Further, non-participation may stunt the sample size and hence reduce statistical power, which in turn means that clinically important effects may go undetected. Finally, there are associated pragmatic difficulties, for example non-participation may increase the cost and resources spent per subject to reach the necessary sample size [2].

The Geelong Osteoporosis Study (GOS) is a large population-based prospective cohort study, initiated in 1993 for women, and in 2001 for men, in order to study the prevalence of osteoporosis, describe age-related changes in bone mineral density and characterize the risks for osteoporosis and fracture [3]. The male and female cohorts have been shown as representative of the broader Caucasian Australian population [3], however, understanding reasons for non-participation, and any associated sex-differences in those reasons, will further inform the strong recruitment process employed by the GOS.

Few studies have examined non-participation in epidemiological research [4]. Existing evidence suggests that the main barriers to participation may include indifference to the subject matter of the study, and demanding lifestyles in a climate of high requests for study participation [1]. In a systematic review of non-participation in randomized controlled trials, a general distrust of hospitals and the medical industry, and a fear of the unknown were also identified as barriers [5]. However, neither of these studies examined potential sex-differences amongst reasons for non-participation. We aimed to investigate reasons for non-participation at the baseline recruitment stages of the GOS and whether they differed by sex. This will inform future recruitment strategies used by researchers undertaking population-based health studies.

## Methods

We examined the baseline recruitment data for the male and female cohort of the GOS, where the recruitment process had randomly selected potential participants from defined age-strata from the Commonwealth electoral rolls encompassing the Barwon Statistical Division (BSD), a region in south-eastern Australia. As voting in Australia is compulsory for residents aged at least 18 years, the electoral roll provides a comprehensive listing of all adult residents and an ideal sampling frame for population-based epidemiological studies. The men were recruited 2001–06 and the women were recruited approximately a decade earlier, in 1993–97. Age-stratified samples were recruited using the same structure for both sexes: approximately 100 individuals were recruited for each 5-year age-group 20–24, 25–29, 30–34, 35–39, 40–44, 45–49, 50–54, 55–59, 60–64 and 65–69 years, and approximately 200 individuals for each of the age groups 70–79 years and 80 years and older. The inclusion criterion was that the individual was listed on the electoral roll for the BSD; if individuals were unable to provide written consent, they were excluded. There was no screening on the basis of exposure to medications or diseases (see [3] for more detailed description).

Of the potential 1,938 women eligible to participate, 444 (22.9%) declined to participate. Of the potential 2,296 men who were eligible to participate, 756 (32.9%) declined. The total number of non-participants was n=1,200 (37% women), and were thus included in this analysis.

During baseline recruitment for the GOS, various phases were employed. Initially, letters of invitation were mailed to the aforementioned randomly selected group of individuals which provided information about the purpose of the study, requirements of participants (clinical tests, blood collection and completion of questionnaires), and advised of the potential time-investment associated with participation and the location of the study centre. Participants were contacted by or requested to contact the research centre by telephone to arrange an appointment for their baseline assessment. Follow-up letters were dispatched at one and two monthly intervals to those who had not yet responded. After attempts to make contact, non-participants were coded according to (i) the reason for non-participation (where known), and (ii) sex.

For this current analysis, reasons for non-participation were pooled on the basis of common underlying themes, and formed seven groups:

(i) personal reason/disinterest (disinterested / can't be bothered /does not want to; personal reasons; no wish to uncover medical problem; invasion of privacy; interference from family; religious / philosophical reasons);

(ii) frailty/inability to cope with or understand the study (frailty/infirmity or too old; other illness; unable to cope with involvement due to old age; cancer; illness associated with fracture; unable to understand the study due to old age);

(iii) time constraints (too busy; work commitments; home commitments);

(iv) undeterminable reason (no reason given; repeated failure to keep appointment);

(v) reluctance over medical testing (avoiding unnecessary tests; avoiding further exposure to x-rays; fear of hospitals; fear of needles (venipuncture));

(vi) language-related issues;

(vii) travel-related issues (too far to travel).

The Pearson’s chi-square test without continuity was used to identify whether there was an overall difference between the sexes regarding reasons for non-participation. Our interpretation of the chi-squared test was based on each group’s contribution to the test statistic. Binary logistic regression analysis was also used to compare sex differences in reasons for non-participation. Statistical analyses were performed using Minitab (Version 15; Minitab, State College, PA). Provision of reason for an individual’s non-participation formed implied consent to utilize the response. The study protocol was approved by the Barwon Health, Human Research Ethics Committee.

## Results

**Figure 1:**
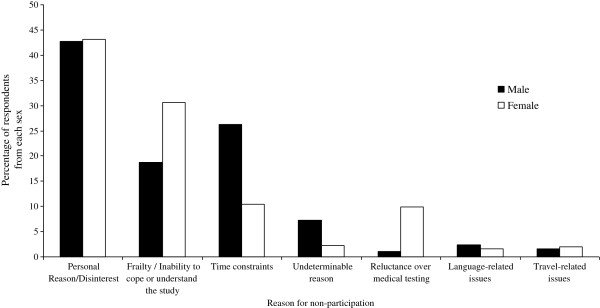
Reasons for non-participation at baseline recruitment of the Geelong Osteoporosis Study (GOS), stratified by sex.

The most frequent reason for non-participation, for both men (42.7%) and women (43.2%), was personal reason/disinterest The next most common reasons for non-participation (in descending order) were: time constraints and frailty/inability to cope with the study for men; and frailty/inability to cope with the study, time constraints, and reluctance over medical testing for women (Table 1 and Figure 1). Overall, there were sex-differences in the reasons provided for non-participation (p < 0.001); apparent differences related to time constraints (men 26.3% vs. women 10.4%), frailty/inability to cope with or understand the study (men 18.7% vs. women 30.6%), and reluctance over medical testing (men 1.1% vs women 9.9%). No sex-differences were observed for non-participation relating to personal reasons/disinterest and language- (men 2.4% vs women 1.6%) or travel-related issues (men 1.6% vs. women 2.0%). Furthermore, reasons for non-participation were undeterminable for a relatively small proportion of people (men 7.3% and women 2.3%).

**Table 1:** Reasons for non-participation (n=1,200, 37% women) at baseline recruitment of GOS, stratified by sex

**Group**	**Subgroup**	**Men n=756**	**Women n=444**	**Total n=1200**
Personal Reason/ Disinterest	Disinterested / can't be bothered /does not want to	299 (39.6)	162 (36.5)	461 (38.4)
	Personal reasons	15 (2.0)	10 (2.3)	25 (2.1)
	No wish to uncover medical problem	6 (0.8)	10 (2.3)	16 (1.3)
	Invasion of privacy	2 (0.3)	3 (0.7)	5 (0.4)
	Interference from family	0 (0.0)	5 (1.1)	5 (0.4)
	Religious / philosophical reasons	1 (0.1)	2 (0.5)	3 (0.3)
	**Overall**	**323 (42.7)**	**192 (43.2)**	**515 (42.9)**
	**Contribution to chi square**	**0.01**	**0.01**	
Frailty / Inability to cope with or understand the study	Frailty/infirmity or too old	68 (9.0)	50 (11.3)	118 (9.8)
	Other Illness	54 (7.1)	42 (9.5)	96 (8.0)
	Unable to cope with involvement due to old age	10 (1.3)	28 (6.3)	38 (3.2)
	Cancer	2 (0.3)	13 (2.9)	15 (1.3)
	Illness associated with fracture	4 (0.5)	1 (0.2)	5 (0.4)
	Unable to understand the study due to old age	3 (0.4)	2 (0.5)	5 (0.4)
	**Overall**	**141 (18.7)**	**136 (30.6)**	**277 (23.1)**
	**Contribution to chi square**	**6.44**	**10.96**	
Time constraints	Too busy	97 (12.8)	33 (7.4)	130 (10.8)
	Work commitments	93 (12.3)	5 (1.1)	98 (8.2)
	Home commitments	9 (1.2)	8 (1.8)	17 (1.4)
	**Overall**	**199 (26.3)**	**46 (10.4)**	**245 (20.4)**
	**Contribution to chi square**	**12.92**	**21.99**	
Undeterminable reason	No reason given	39 (5.2)	4 (0.9)	43 (3.6)
	Repeated failure to keep appointment	16 (2.1)	6 (1.4)	22 (1.8)
	**Overall**	**55 (7.3)**	**10 (2.3)**	**65 (5.4)**
	**Contribution to chi square**	**4.82**	**8.21**	
Reluctance over medical testing	Avoiding unnecessary tests	3 (0.4)	19 (4.3)	22 (1.8)
	Avoiding further exposure to x-rays	5 (0.7)	14 (3.2)	19 (1.6)
	Fear of Hospitals	0 ( − )	9 (2.0)	9 (0.8)
	Fear of needles (venipuncture)	0 ( − )	2 (0.5)	2 (0.2)
	**Overall**	**8 (1.1)**	**44 (9.9)**	**52 (4.3)**
	**Contribution to chi square**	**18.71**	**31.86**	
Language-related issues	Language problems: **Overall**	**18 (2.4)**	**7 (1.6)**	**25 (2.1)**
	**Contribution to chi square**	**0.32**	**0.55**	
Travel-related issues	Too far to travel (living within study region)	11 (1.5)	9 (2.0)	20 (1.7)
	Too far to travel (has moved out of the region)	1 (0.1)	0 (− )	1 (0.1)
	**Overall**	**12 (1.6)**	**9 (2.0)**	**21 (1.8)**
	**Contribution to chi square**	**0.11**	**0.20**	
**Total**		756 (100)	444 (100)	1,200

Pearson’s chi-square = 117.101, DF = 6, p value < 0.001.

Data are shown as n (%).

Using the group who gave personal reasons or disinterest as the reference, men were more than twice as likely as women to be a non-participant because of limited time (Table 2). In contrast, men were considerably less likely than women to state they were too frail to participate (OR = 0.62, 95%CI 0.46-0.83) or they felt reluctant to have medical tests (OR = 0.11, 95%CI 0.05-0.23).

**Table 2:** Odds ratios (and 95% confidence intervals, CI) expressing the likelihood of being male for each group of reasons for non-participation using personal reason/disinterest as the reference category

**Group**	**Odds Ratio**	**95% Confidence Interval**
Personal reason/Disinterest	1.00	-
Frailty	0.62	0.46, 0.83
Time constraints	2.57	1.78, 3.71
Undeterminable reason	3.27	1.63, 6.56
Reluctance over medical testing	0.11	0.05, 0.23
Language-related issues	1.53	0.63, 3.73
Travel-related issues	0.79	0.33, 1.92

## Discussion

We observed that a greater proportion of men declined participation in the baseline GOS recruitment compared to women. For men, the most common reasons for non-participation, in descending order were: (i) personal reason/disinterest, (ii) time constraints and (iii) frailty/inability to cope with the study requirements. For women, the most common reasons for non-participation in descending order were: (i) personal reason/disinterest, (ii) frailty/inability to cope with the study, (iii) time constraints, and (iv) reluctance over medical testing. Men were more likely to decline participation due to time constraints than women. Women were more likely to decline due to reasons associated with frailty and due to reluctance over medical testing, compared to men.

Our observation that men had an increased propensity to decline participation compared to women, is consistent with other studies [6,7]. Interestingly, we observed no sex-difference in the level of disinterest; furthermore, we can confirm that disinterest was not borne out of religious/philosophical objections, family interference, privacy concerns, or a fear of uncovering a medical problem (subgroups that were collapsed to form the personal reason/disinterest group). However, evidence exists to suggest that people who experience symptoms relevant to the health conditions under investigation are more likely to take part in studies than those who do not have those symptoms [1,8]. Given that osteoporosis often remains undetected until after a fragility fracture occurs [9], it is plausible that non-participation related to disinterest may be due to a lack of awareness of the disease. Osteoporosis has also been identified as a condition with ‘low salience’ among the lay and medical communities – there exists a level of ambivalence and unconcern about osteoporosis in individual patients and the general population [10,11]. Other factors that hampered interest may have been that certain attributes of the study were not salient or appealing enough to provide motivation to participate [12]; people may not see a personal gain from participating or feel disillusioned with scientific research on the whole [13]. Thus, highlighting personal relevance during the recruitment phase of a study may help combat antipathy towards research participation.

Women were more likely than men to decline participation due to frailty, or the inability to cope with requirements of the GOS study. The recruitment of elderly individuals to epidemiological studies has obvious difficulties [14,15]. A number of published studies that examined frailty-related reasons for non-participation suggested that women may be impeded by personal illness more so than men [16].

Compared to men, we also observed women to be more likely to decline participation due to reluctance over medical testing. A 2007 study reported that women may have a stronger perception of risks and harms involved with epidemiological research compared to men [17]. This suggestion offers a plausible explanation for our observations, especially given that participation in the GOS required a number of clinical examinations. Furthermore, reluctance was not due to an avoidance of confronting medical issues, but rather due to concerns about possible stress and adverse effects of the battery of clinical examinations proposed.

The final key reason for non-participation was related to time constraints, whereby men were more likely to report limited time compared to women. To minimise problems associated with availability due to work commitments, the GOS offered potential participants appointment times after hours and at weekends. Time constraints have consistently been reported by other studies as a barrier to participation [6,18].

This study has some notable strengths. First, the GOS re-contacted non-responders, whereby those who did not respond to the initial invitation were followed-up. A problem that is common to studies where mail surveys are utilised is that often there is no attempt made to ascertain whether non-response was due to the letter not being received, or because individuals have consciously chosen not to participate [18,19]. Second, the GOS did not employ ambiguous categories to define reasons for non-participation, but rather explored precise reasons to improve clarity. This study also has some limitations. We are unable to comment on age-related reasons for non-participation, due to these data being unavailable. Furthermore, the female and male cohorts of the GOS were recruited 10 years apart, so the observed sex-differences in reasons may reflect a period effect, whereby the consequences of influences may vary with time.

## Conclusions

In conclusion, we observed sex-differences in reasons for non-participation in the GOS; frequencies for reasons relating to frailty, time constraints, and reluctance over medical testing were not the same for men and women. Our findings also suggest that the GOS recruitment process may have limited the potential for sex-bias with regards to disinterest, language and travel issues. This study highlights the importance of stimulating interest in the aims of the research project during recruitment, which may be achieved by the early establishment of rapport to promote participation. Furthermore, targeted attention to providing information about the safety and risks involved in clinical tests may aid people’s decisions about whether or not to participate. We suggest that this exploration of sex-differences in reasons for non-participation may help future researchers to enhance specific recruitment strategies and protocols, depending on their target population.

## Competing interests

The authors have no competing interests to declare.

## Authors’ contributions

SLB and JAP conceived of and designed the study. SM performed the statistical analysis and drafted the manuscript. JAP helped collect the data and takes responsibility for the integrity of the study. SLB, HG and JAP critically revised the manuscript. All authors read and approved the final manuscript.
